# Notes and Formulæ

**Published:** 1889-11

**Authors:** 


### PRACTICAL NOTES AND FORMULÆ.

**Eczema, Chronic.**—

| | |
|---|---|
| Take of sulphide of arsenic, | 1 grain. |
| Beta-naphthol, | 24 grains. |

Mix and divide into twelve pills.

Dose. One pill three times a day.—*Peoria Med. Monthly.*

**Powder Stains on the Skin.**—The bluish-black spots produced by gunpowder may be removed by painting with the solution:

Biniodide of ammonium,
Distilled water, equal parts.

Then with dilute hydrochloric acid to reach the tissues more deeply affected.—*Ib.*

**For Summer Diarrhœa.**—

| | | |
|---|---|---|
| ℞ | Tinct. hydrastls, | 1 ounce. |
| | Potas. carbonat., | 1 drachm. |
| | Syr. rhei aromat, | 7 ounces. |

M. Sig. Half-ounce every four hours.—*Ib.*

**Menthol in Pruritus.**—

| | | |
|---|---|---|
| ℞ | Menthol, | 22 to 36 grains. |
| | Alcoholis, | 1½ ounces. |

M. ft. lotio.

| | | |
|---|---|---|
| ℞ | Menthol, | 36 grains. |
| | Olive oil, | 2 to 3 drachms. |
| | Lanolin, | 1½ ounces. |

M. ft. unguent,

| | | |
|---|---|---|
| ℞ | Menthol, | 48 grains. |
| | Balsam of Peru, | 96 grains. |
| | Benzoated oxide of zinc, | |
| | Lanolin, pure, | ää 2 ounces. |

M. ft. unguent. For use in scabies.—*Ib.*

**Laxative Powder.**—

| | | |
|---|---|---|
| ℞ | Powdered anethum, | |
| | Powdered senna, | |
| | Sublimed sulphur | ää 6 grammes. |
| | Powdered cream of tartar | 2 " |
| | Powdered licorice | 8 " |
| | Powdered sugar | 25 " |

M. Sig. Teaspoonful to a dessertspoonful to be taken at night in a little water.

**Ophthalmic Migraine** (Piechaud).—

| | | |
|---|---|---|
| ℞ | Syrup of ether | 60 grammes. |
| | Antipyrin | 3 " |
| | Citrate of caffein | 0 gr. 20. |
| | Cocaine hydrochlorate | 0 gr. 20. |

M. Sig. A tablespoonful every three hours until relieved.
—*Revue de Therapeutique.*

**Nasal Catarrh** (Garageorgiades).—

| | | |
|---|---|---|
| ℞ | Pulverized iodoform, | |
| | Powderd acacia | ää 2 grammes. |
| | Hydrochlorate of cocaine | 0 gr 10. |

M. Sig. Use as snuff.—*Times and Register.*

**Laxative Pills.**—

| | | |
|---|---|---|
| ℞ | Sulphate of iron, | |
| | Extract of aloes, | |
| | Extract of hyoscyamus | ää 1 gramme. |
| | Alcoholic extract of nux vomica, | 0.15 centigrammes. |
| | Make fifteen silver-coated pills. | |

M. Sig. One at night.

—*La Normandy Medicale.*

**Treatment of Pruritus Senilis.**—M. E. Besnier offers the following as an effective treatment in this distressing affection (*Faance Medicale*): 1° Starch baths. 2° Apply at night hot water (110° F.) to which the following has been added:

| | |
|---|---|
| Carbolic acid | 4 parts. |
| Aromatic vinegar | 200 parts. |

3° Then apply one of the following powders:

| | | |
|---|---|---|
| ℞ | Bismuth. salicylat | ʒ ij. |
| | Amyli | ʒ ix. |

M.

This powder should be lightly applied.—*Med. Progress.*

**Treatment for Excessive Menstruation** (Rheinstadter).—

| | | |
|---|---|---|
| ℞ | Ergotine dialysed | 10 grammes |
| | Distilled water | 70 " |
| | Glycerin | 20 " |
| | Salycilic acid | 0 gr. 2 |

M. Sig. One teaspoonful diluted with three tablespoonfuls of water, to be injected into the rectum once a day after a stool.—*Ex.*

**Ulceration of the Ear.**—A case of chronic ulceration of the ear, emitting a fetid discharge, was treated by the following:

| | | |
|---|---|---|
| ℞ | Acid nitrici, | 10 drops. |
| | Aquæ, | 8 ounces. |

A teaspoonful of this mixture thrown into the ear two or there times a day, put a stop to the disease in less than a week.

